# Evaluating statistical models for overdispersed multiomics data: a multiplex immunofluorescence case study

**DOI:** 10.1093/aje/kwag127

**Published:** 2026-06-15

**Authors:** Claire E Thomas, Evertine Wesselink, Yasutoshi Takashima, Jeroen R Huyghe, Daniel D Buchanan, Robert C Grant, Andressa Dias Costa, Tomotaka Ugai, Shuji Ogino, Jonathan A Nowak, Ulrike Peters, Amanda I Phipps, Li Hsu

**Affiliations:** Public Health Sciences Division, Fred Hutchinson Cancer Center, Seattle, WA, United States; Division of Molecular Pathology, Netherlands Cancer Institute-Antoni van Leeuwenhoek Hospital, Amsterdam, The Netherlands; Department of Medical Oncology, Dana-Farber Cancer Institute, Boston, MA, United States; Public Health Sciences Division, Fred Hutchinson Cancer Center, Seattle, WA, United States; Colorectal Oncogenomics Group, Department of Clinical Pathology, Melbourne Medical School, The University of Melbourne, Parkville, Australia; Collaborative Centre for Genomic Cancer Medicine, The University of Melbourne, Victorian Comprehensive Cancer Centre, Parkville, Australia; Genomic Medicine and Family Cancer Clinic, The Royal Melbourne Hospital, Parkville, Victoria, Australia; Division of Medical Oncology and Hematology, Princess Margaret Cancer Centre, University Health Network, Toronto, Ontario, Canada; Department of Medical Oncology, Dana-Farber Cancer Institute and Harvard Medical School, Boston, MA, United States; Program in MPE Molecular Pathological Epidemiology, Department of Pathology, Brigham and Women’s Hospital and Harvard Medical School, Boston, MA, United States; Department of Epidemiology, Harvard T.H. Chan School of Public Health, Boston, MA, United States; Program in MPE Molecular Pathological Epidemiology, Department of Pathology, Brigham and Women’s Hospital and Harvard Medical School, Boston, MA, United States; Department of Epidemiology, Harvard T.H. Chan School of Public Health, Boston, MA, United States; Broad Institute of MIT and Harvard, Cambridge, MA, United States; Institute of Science Tokyo, Tokyo, Japan; Program in MPE Molecular Pathological Epidemiology, Department of Pathology, Brigham and Women’s Hospital and Harvard Medical School, Boston, MA, United States; Department of Oncologic Pathology, Dana-Farber Cancer Institute, Boston, MA, United States; Public Health Sciences Division, Fred Hutchinson Cancer Center, Seattle, WA, United States; Department of Epidemiology, University of Washington, Seattle, WA, United States; Public Health Sciences Division, Fred Hutchinson Cancer Center, Seattle, WA, United States; Department of Epidemiology, University of Washington, Seattle, WA, United States; Public Health Sciences Division, Fred Hutchinson Cancer Center, Seattle, WA, United States; Department of Biostatistics, University of Washington, Seattle, WA, United States

**Keywords:** count data modeling, overdispersion, zero inflated data, negative binomial, ordinal logistic regression, simulation

## Abstract

Multiomic data analysis poses statistical challenges. We evaluated statistical models for our multiplex immunofluorescence study of T cell subset densities in colorectal cancer. Using 1235 cases, we compared 7 models—ordinal logistic regression, Poisson, quasi-Poisson, quadratic negative binomial (NB), linear NB, zero-inflated NB, and hurdle NB models—assessing associations with a strong (microsatellite instability, MSI) and a weak (calcium intake) exposure. Simulation studies assessed type I error and power. Effect estimates were generally consistent for the strong exposure (MSI) but varied for the weaker exposure (calcium). Simulations revealed inflated false-positive rates for the Poisson and NB-based models, including quadratic NB, zero-inflated, and hurdle, but not for ordinal logistic regression or the linear NB model. The quasi-Poisson model showed modest inflation of low *P*-values, but the overall *P*-value distribution remained approximately uniform under the null. Ordinal logistic, linear NB, and quasi-Poisson models achieved the best or near-best power across a range of zero proportions, dispersion levels, and distributions. The ordinal logistic, linear NB, and quasi-Poisson models are useful and robust options for epidemiologic analyses of overdispersed, right-skewed multiomic data with a nontrivial proportion of zero counts.

## Introduction

Multiomic data are increasingly integrated into epidemiologic studies to reveal how biological and environmental exposures impact disease risk and progression. Advances in generating high-resolution, high-dimensional molecular data, create valuable opportunities to leverage biobanked samples from epidemiologic studies^1^^,^^2^ to facilitate this multiomic work. For example, spatial proteomics, spatial transcriptomics, single-cell RNA sequencing, mass spectrometry, and targeted sequencing platforms generate multiomic data that illuminate the molecular pathways through which exposures, such as the microbiome, tumor immune microenvironment (TIME), gene expression, protein abundance, and per- and polyfluoroalkyl substances influence health.^2-5^ However, these data are often overdispersed, right-skewed, and prone to high levels of zero counts or sparsity, leading to challenges for modeling and integrating these complex data sources.^6-11^

The granularity of multiomic data presents a statistical challenge: subdividing heterogenous data into finer groups increases the percentage of samples with zero values for specific subsets. Negative binomial (NB) models have previously been suggested for overdispersed, right-skewed count data with some level of zero counts^11-13^; however, their validity in such settings has also been questioned.^14^ Zero-inflated and hurdle NB models have also been suggested for data with a large percentage of zero counts.^11^^,^^15^ Given substantial percentages of zeros and skewness in multiomic data, careful statistical modeling is vital. Model misspecification can lead to erroneous conclusions, underscoring the need for a rigorous comparison of statistical approaches.

The goal of this paper is to apply and compare various statistical models commonly used for overdispersed and right-skewed data with high levels of zero counts. We examine multiplex immunofluorescence (mIF) data from tissue microarrays (TMAs), characterizing abundance and density of T cell subsets in colorectal cancer (CRC). mIF assays enable detailed characterization of the TIME and have been critical for discovering different immune phenotypes and spatially resolved cellular interactions.^16^^,^^17^ As technology has improved, mIF and multiplex immunohistochemistry (IHC) have become reliable high-throughput methods, allowing identification of multiple immune and tumor cell populations within the same slide while preserving spatial biology of the TIME.^18^^,^^19^ The statistical challenges of multiomic data are illustrated in this example in the use of data generated from TMAs. TMAs are a useful tool in large-scale epidemiologic studies that allow for high throughput of multiple formalin-fixed paraffin-embedded (FFPE) sample cores from multiple patients with reduced batch effects, time, and cost,^19^^,^^20^ though their limited tissue sampling area may underrepresent biomarker heterogeneity. The combination of improved technology identifying many cell subsets as well as the utilization of TMAs with a relatively small tumor tissue area compared to whole slides exacerbate the impact of zero counts, particularly for rare immune cell subtypes.

As two case studies, we assessed the association between T cell densities and two exposures: one strongly associated with immune profile in CRC (microsatellite instability, MSI status)^21^ and one that exhibits a weak association (total calcium intake).^22^ We then conducted simulation studies to evaluate model performance. Finally, we revisited our original analysis to identify findings vulnerable to model misspecification, demonstrating the importance of appropriate model choice in drawing valid inferences from this complex data source.

## Methods

### Study population, MSI, and calcium

We used data from three Genetics and Epidemiology of Colorectal Cancer Consortium cohorts with tumor immune profiling data^21^: the Ontario Family Colon Cancer Registry (OFCCR),^23^ the Nurses’ Health Study (NHS),^24^ and the Health Professionals Follow-up Study (HPFS).^24^ In total, 1315 participants had tumor immune profiling data. TMA cores include both epithelial and stromal tissue regions. This analysis focuses on the epithelial tissue area, where the range of zero-percentage for various subsets is broader, allowing for a more comprehensive evaluation of different statistical models. After excluding individuals with missing MSI and calcium exposure data, 1235 and 936 individuals remained, respectively. MSI status was assessed from FFPE tumor tissue using study-specific protocols,^21^^,^^23^^,^^25-30^ with NHS and HPFS^28^ using polymerase chain reaction (PCR) and OFCCR,^23^^,^^29^ employing both PCR and IHC methods. For harmonization, MSI status was categorized as MSI-high or non-MSI-high (MSI-low/MSS, microsatellite stable) for analysis.^31^ Total calcium intake was derived from study questionnaires and harmonized across studies, combining dietary calcium intake, which was measured using semiquantitative food frequency questionnaires, and supplemental calcium intake, which was measured in number of pills a day.^32-35^ All participants gave written informed consent and studies were approved by their respective Institutional Review Boards. Further details are previously published.^21^^,^^22^^,^^36^

### T cell profiling

We profiled the *in situ* T cell landscape of CRC using an mIF panel as described previously.^21^ TMA construction using standard methods has been described in detail elsewhere.^37^ Assays were conducted on TMA sections using a customized 9-plex panel that included antibodies targeting CD3, CD4, CD8, PTPRC (CD45RO, CD45RA), FOXP3, MKI67 (Ki-67), as well as a KRT (keratin, pan-cytokeratin) antibody to identify tumor cells and a di-(4-amidinophenyl)-1H-indole-6-carboxamidine stain to identify nuclei. This marker panel was validated against traditional chromogenic IHC. Using a machine learning-based cell phenotyping algorithm trained specifically for CRC^38^^,^^39^ (inForm 2.6.0, Akoya Biosciences, Marlborough, MA, United States), T cell densities were quantified within each TMA core, allowing identification of naive, memory, and regulatory helper (CD3^+^CD4^+^) T cells, naive, memory, and regulatory cytotoxic (CD3^+^CD8^+^) T cells, double-negative naive and memory T cells, and CD3^–^ immune cells based on marker co-expression, resulting in 9 distinct subsets. T cell subset definitions as defined by marker co-expression have been previously published.^21^^,^^36^ The total tissue area of each core was divided into epithelial, stromal, and empty regions (predominantly glandular lumina) based on pan-cytokeratin expression using a machine learning-based tissue classification algorithm. For each CRC case, we used the count or density of each T cell subset in tumor epithelial regions averaged across multiple TMA cores.

### Data analysis

We investigated several statistical modeling approaches for analyzing mIF data, each with its own advantages and disadvantages. We focused on the following nonoverlapping subsets of epithelial T cells: naive, memory, and regulatory CD3^+^CD4^+^ T cells; naive, memory, and regulatory CD3^+^CD8^+^ T cells; naive and memory CD3^+^CD4^–^CD8^–^ double negative T cells, and CD3^–^ immune cells. The strengths, limitations, and interpretation of each model are summarized in Table S1. All multivariable models were adjusted for age, sex, tumor location (proximal colon, distal colon, rectum, and missing), and study batch (NHS and HPFS, OFCCR). All analyses were performed using R, version 4.4.0 (R Foundation for Statistical Computing, Vienna, Austria) software.

#### Ordinal logistic regression

Ordinal logistic regression models are widely used in epidemiological research.^21^^,^^40^ These models examine associations between an exposure and an ordinal outcome variable (eg, low, medium, and high; tertiles; and quartiles). The ordinal logistic regression model assumes that the outcome variable has a natural order and the proportional odds of being in a higher category is constant across thresholds. We applied these models to densities (T cells per mm^2^ tissue area) of epithelial T cell subsets, categorized depending on the percentage of zeros (Table 1). Subsets with less than 25% zeros were divided into quartiles, subsets with greater than 25% and less than 60% zeros were divided into tertiles, and subsets with greater than 60% zeros were categorized as binary (zero vs non-zero). Odds ratios (OR) and their 95% CIs were estimated. The polr function within the MASS R package was used for ordinal logistic regression estimation.

**Table 1:** Descriptive statistics of T cell subsets in MSI dataset (*N* = 1235).

**Epithelial tissue area immune subset density**	**Density zeros *N* (%)**	**Density mean**	**Density variance**	**Overdispersion (variance > mean)**	**Ordinal logistic regression categorization** ^**a**^
**High percent zeros**					
Double negative naive	1187 (96)	0.2	4.5	Yes	Binary
Double negative memory	1020 (83)	1.2	20	Yes	Binary
**Medium percent zeros**					
CD3^+^CD4^+^ naive	605 (49)	18	10 384	Yes	Tertiles
CD3^+^CD4^+^ regulatory	579 (47)	11	1589	Yes	Tertiles
CD3^+^CD8^+^ naive	380 (31)	71	63 257	Yes	Tertiles
CD3^+^CD8^+^ regulatory	355 (29)	70	49 342	Yes	Tertiles
**Low percent zeros**					
CD3^+^CD4^+^ memory	288 (23)	48	8101	Yes	Quartiles
CD3^+^CD8^+^ memory	271 (22)	65	33 439	Yes	Quartiles
CD3^–^ immune cells	5 (<1)	489	284 683	Yes	Quartiles

Abbreviation: MSI, microsatellite instability.

Double negative = CD3^+^CD4^–^CD8^–^.

a Categorization for ordinal or binary logistic regression modeling. Categories were assigned based on the larger percentage of zeros, where less than 25% zeros were categorized as quartiles, greater than 25% and less than 60% zeros were categorized to tertiles, and greater than 60% zeros were categorized as binary. Categorizations were consistent across MSI and calcium exposure datasets.

#### Poisson and quasi-Poisson count models

Poisson and quasi-Poisson regression models are also commonly used to analyze count outcomes.^41^ These models estimate associations between an exposure and the expected number of events (eg, cell counts) within a defined area or time. The Poisson model assumes that counts follow a Poisson-type distribution and are modeled using a log-link, allowing coefficients to be interpreted as incidence rate ratios (IRRs). Under this model, the variance equals the mean (equidispersion). In comparison, the quasi-Poisson model assumes the same mean structure as the Poisson model but relaxes the variance assumption by allowing it to be proportional to the mean, thereby accommodating over- or underdispersion. All models used counts as the outcome and included tissue area as an offset term to account for differences in TMA core size (ie, modeling rates per mm^2^). The glm function in R with family = Poisson or family = quasi-Poisson was used for model estimation.

#### NB models

The NB^14^ model is a generalization of the Poisson model, which relaxes the restrictive assumption that the variance and mean are equal,^12^ making it potentially more appropriate for analyzing overdispersed count data.^11-13^ Given that the T cell subsets identified as single-cell counts were highly right-skewed and overdispersed, NB models could provide a better fit. Previous work on mIF data suggests that the NB model is appropriate when fewer than 50% of cell type counts are zeros, supporting its use in our data.^11^ NB models were applied using counts with an offset term for tissue area (cells per mm^2^ tissue area). We considered 2 forms of NB models: the NB model with a quadratic mean–variance relationship (quadratic NB), and the type II NB model as defined in the GAMLSS framework,^42^ with a linear mean–variance relationship (linear NB). We chose to refer to these models explicitly by their variance structures (quadratic or linear) to avoid the conflicting “Type I” and “Type II” naming conventions found across different software packages and statistical literature. IRR and 95% CIs were estimated for the associations between the exposure and T cell subsets. The glm.nb function within the MASS R package was used for the quadratic NB model, and the gamlss function with family = NBII within the GAMLSS package developed by Stasinopoulos and Rigby^42^ was used for the linear NB model.

#### Zero-inflated NB and hurdle NB

The zero-inflated negative binomial (ZINB) model was developed for count data with excess zeros and overdispersion.^15^ It consists of 2 components: (1) a zero-inflation component that estimates the probability of belonging to the non–at-risk (structural zero) group and (2) a count component that models counts using an NB distribution. The ZINB model is powerful for complex data with many zeros but requires careful interpretation and validation. In our analysis, we included all covariates and an offset term for tissue area in the count component but only included tissue area for the binary (zero-inflation) component. Given instability observed in the binary component, we focused inference for exposure of interest on the count component.

The hurdle-NB model is a related approach that also includes a binary logistic regression component for distinguishing zeros vs non-zeros. However, instead of modeling counts with a standard NB distribution as in ZINB, it uses a truncated NB model, where the model is truncated at zero.^15^ For the hurdle model, we included covariates and offset terms in both the binary and count components and report estimates for the association of the exposures of interest from each component. ORs and 95% CI for binary models and IRR and 95% CI were estimated for both ZINB and hurdle NB count models using the zeroinfl and hurdle functions in the pscl R package.

## Simulation study

### Type I error

We conducted a simulation study to examine the type I error of all modeling approaches considered: ordinal logistic regression, Poisson, quasi-Poisson, quadratic NB, linear NB, ZINB, and hurdle NB, using our real data as the basis for simulation. Specifically, we permuted the exposure variable in our real-world observed data to break any associations between exposure and outcome such that the resulting *P*-value distributions should follow a uniform distribution under the null hypothesis. We generated 10 000 permuted datasets.

Models were applied to all 9 T cell subsets, excluding ZINB and hurdle for CD3^–^ immune cells due to low-zero instability. All simulation models were fit without covariates but retained offset terms where applicable for count models. The ZINB model was conducted in a manner consistent with the main analyses, with the permuted exposure of interest included only in the count component, not in the binary component. When an excessive number of false positives were observed, we further stratified the results by high, medium, and low percentages of zero counts, to assess the impact of zero counts on the model performance.

### Power

We next examined power, focusing on models that demonstrated reasonably well-controlled type I error in our real data-based simulation: ordinal logistic regression, quasi-Poisson, and linear NB. We also included the quadratic NB model, mainly to compare with the linear NB model. Specifically, we selected 3 representative T cell subsets to serve as templates for data generation: (1) high percentage of zeros (double negative memory), (2) moderate percentage of zeros (CD3^+^CD4^+^ regulatory), and (3) low percentage of zeros (CD3^+^CD8^+^ memory). For each subset, we fit an NB model with MSI status as the exposure of interest and tissue area as offset term. Using the estimated parameters from this model, along with the observed MSI status and tissue area, we generated counts from an NB distribution, while systematically varying the regression coefficient, *β* (beta), for the association between MSI status and the outcome, as well as the dispersion parameter, *θ* (theta). The regression coefficient varied from 0 to 1 with an increment of 0.05, and 3 values of the dispersion parameter were considered (0.5, 1, and 2). For each combination of regression coefficient and dispersion parameter, a total of 10 000 datasets were generated. We generated these simulations under both linear and quadratic NB distributions to compare results.

## Results

**Figure 1:**
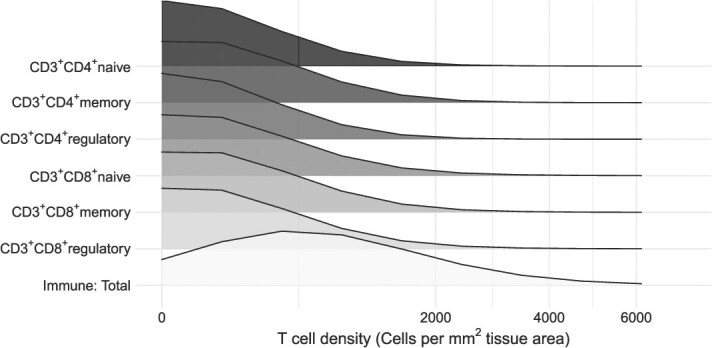
Overdispersed, right-skewed distributions of specific T cell subsets and total immune cell densities in epithelial areas of colorectal cancer (CRC) tumors (*N* = 1235).

Characteristics about participants in our study population have been published previously.^21^^,^^22^ Briefly, the mean age at diagnosis (SD) was 67 (9) years, and proportions of men and women were roughly equal. The majority of tumors were MSI-low/MSS (82%).^21^ Median total calcium intake was 1041 mg/day (IQR, 757-1349). Densities of T cell subsets in the epithelial tissue area were overdispersed and right-skewed (Figure 1 and Table 1). Percentage of zero counts varied by T cell subset, where double negative naive and memory T cells were high with more than 80% zeros, 4 subsets had a medium level of zeros ranging from 29% to 49%, and 3 subsets, CD3^+^CD4^+^ and CD3^+^CD8^+^ memory T cells and CD3^–^ immune cells, had low levels of zeros (<1%-23%; Table 1). Percentages of zeros were similar in the MSI and calcium datasets (data not shown).

### Initial model comparisons

Results related to the biological question for the association between MSI status and T cell densities have been published previously, utilizing ordinal logistic regression.^21^ Across all models examined, MSI-high status was generally associated with higher levels of epithelial tissue area T cell densities compared to MSI-low/MSS tumors (Table 2). Ordinal logistic, Poisson, quasi-Poisson, quadratic NB, and linear NB regression models were largely consistent in directionality and statistical significance, with 2 exceptions: the quasi-Poisson model did not find a significant association between MSI status and CD3^+^CD4^+^ naive T cells, whereas all other models did; and the ordinal logistic and linear NB models did not find a significant association between MSI status and CD3^+^CD4^+^ memory T cells, whereas all other models did. ZINB and hurdle NB models were largely consistent in estimates with other models; however, ZINB models were less stable than hurdle NB models even when modeling the exposure of interest was restricted to the count component alone, with nonconvergence observed for outcomes with high percentages of zeros (Table 2).

**Table 2:** Association between microsatellite instability status (strong exposure) and T cell subset densities utilizing multiple modeling approaches (*N* = 1235).

**Epithelial tissue area immune subset density**	**Ordinal logistic, densities: OR (95% CI)**	**Poisson, count: IRR (95% CI)**	**Quasi-Poisson, count: IRR (95% CI)**	**Quadratic NB, count: IRR (95% CI)**	**Linear NB, count: IRR (95% CI)**	**Zero-inflated NB, count: IRR for count** ^**a**^ **: IRR (95% CI)**	**Hurdle NB, count: OR for binary and IRR for count:** **IRR or OR (95% CI)**
**High percent zeros**							
Double negative naive	**2.41 (1.16, 4.89)**	-	-	-	-	4.17 (NA, NA)	Count IRR: 19.28 (0, Inf) Zeros OR: 3.55 (0.97, 12.97)
Double negative memory	**2.49 (1.68, 3.70)**	**-**	-	-	**-**	4.92 (NA, NA)	**Count IRR: 7.27 (2.21, 23.96) Zeros OR: 2.89 (1.75, 4.78)**
**Medium percent zeros**							
CD3^+^CD4^+^ naive	**1.65 (1.22, 2.22)**	**1.22 (1.13, 1.31)**	1.22 (0.78, 1.87)	**1.65 (1.17, 2.35)**	**1.35 (1.08, 1.68)**	**1.61 (1.14, 2.29)**	**Count IRR: 1.81 (1.05, 3.09)** Zeros OR: 1.37 (0.97, 1.92)
CD3^+^CD4^+^ regulatory	**2.03 (1.51, 2.74)**	**1.63 (1.48, 1.8)**	**1.63 (1.13, 2.34)**	**1.83 (1.34, 2.53)**	**1.62 (1.31, 2.00)**	**1.80 (1.31, 2.47)**	**Count IRR: 1.92 (1.21, 3.05)** **Zeros OR: 1.82 (1.31, 2.54)**
CD3^+^CD8^+^ naive	**3.49 (2.57, 4.75)**	**6.16 (5.95, 6.37)**	**6.16 (4.64, 8.20)**	**4.76 (3.59, 6.40)**	**2.27 (1.91, 2.70)**	**4.69 (3.51, 6.25)**	**Count IRR: 4.35 (2.96, 6.39)** **Zeros OR: 3.84 (2.56, 5.76)**
CD3^+^CD8^+^ regulatory	**3.04 (2.24, 4.13)**	**5.24 (5.06, 5.43)**	**5.24 (4.07, 6.77)**	**4.66 (3.53, 6.24)**	**2.12 (1.79, 2.52)**	**4.60 (3.47, 6.11)**	**Count IRR: 4.49 (3.15, 6.41)** **Zeros OR: 3.11 (2.06, 4.69)**
**Low percent zeros**							
CD3^+^CD4^+^ memory	1.28 (0.96, 1.71)	**1.53 (1.46, 1.61)**	**1.53 (1.19, 1.96)**	**1.59 (1.25, 2.05)**	1.14 (0.96, 1.34)	**1.60 (1.25, 2.05)**	**Count IRR: 1.86 (1.39, 2.49)** Zeros OR: 0.92 (0.63, 1.34)
CD3^+^CD8^+^ memory	**2.82 (2.10, 3.78)**	**3.23 (3.1, 3.36)**	**3.23 (2.48, 4.21)**	**3.63 (2.79, 4.75)**	**1.82 (1.55, 2.14)**	**3.63 (2.78, 4.73)**	**Count IRR: 3.78 (2.64, 5.42)** **Zeros OR: 2.76 (1.78, 4.27)**
CD3 immune cells	**2.10 (1.58, 2.79)**	**1.59 (1.57, 1.62)**	**1.59 (1.39, 1.82)**	**1.55 (1.35, 1.78)**	**1.42 (1.28, 1.57)**	-	-

Abbreviations: Inf, infinity, unstable high value, IRR, incidence rate ratio; NA, non-converging value, NB, negative binomial; OFCCR, Ontario Family Colon Cancer Registry;. OR, odds ratio; ZINB, zero-inflated negative binomial.

Adjusted for age, sex, study batch (Harvard/OFCCR), and cancer site (proximal, distal, rectal, missing).

Bold values indicate *P*-value <0.05.

Cells with dashed lines indicate cases where models were not run because they were unsuitable based on the percentage of zeros (eg, zero-inflated models were skipped for CD3^–^ immune cells, and count models were avoided for subsets with a high percentage of zeros).

Ordinal logistic regression results were previously published in Thomas et al.^21^

a The binary component of the ZINB model only included tissue area as a covariate, and did not model our exposure of interest due to model instability.

Results related to the biological question for the association between calcium intake and T cell densities have been published previously, utilizing ordinal logistic regression.^22^ In contrast to the strong exposure (MSI), associations of T cell subsets with the weak exposure (calcium intake, per quartile increase) varied in statistical significance across models for the association between calcium intake (per quartile increase) and T cell subsets. A higher calcium intake compared to a lower calcium intake was associated with only a few immune subsets, none of them consistent over all models examined (Table 3).

**Table 3:** Association between total calcium intake (weak exposure) and T cell subset densities utilizing \marginpar{text}multiple modeling approaches (*N* = 936).

**Epithelial tissue area immune subset density**	**Ordinal logistic, densities** **OR (95% CI)**	**Poisson, count: IRR (95% CI)**	**Quasi-Poisson, count: IRR (95% CI)**	**Quadratic NB, count: IRR (95% CI)**	**Linear NB, count:** **IRR (95% CI)**	**Zero-inflated NB, count: IRR for count** ^**a**^ **IRR (95% CI)**	**Hurdle NB, count: OR for binary and IRR for count** **IRR or OR (95% CI)**
**High percent zeros**							
Double negative naive	1.25 (0.92; 1.71)	-	-	-	**-**	1.62 (0.84, 3.12)	Count IRR: 1.95 (0.01, 395.83) Zeros OR: 1.44 (0.86, 2.42)
Double negative memory	1.03 (0.87; 1.21)	-	-	-	-	1.02 (0.81, 1.30)	Count IRR: 0.8 (0.52, 1.22) Zeros OR: 1.08 (0.87, 1.34)
**Medium percent zeros**							
CD3^+^CD4^+^ naive	1.01 (0.90; 1.12)	**1.14 (1.11, 1.17)**	1.14 (0.96, 1.35)	1.12 (0.99, 1.28)	1.01 (0.93, 1.10)	1.13 (0.99, 1.28)	**Count IRR: 1.30 (1.07, 1.58)** Zeros OR: 0.98 (0.86, 1.10)
CD3^+^CD4^+^ regulatory	**1.14 (1.03; 1.27)**	**1.16 (1.12, 1.21)**	1.16 (1, 1.35)	1.09 (0.97, 1.23)	1.09 (1.00, 1.18)	1.09 (0.97, 1.23)	Count IRR: 1.02 (0.86, 1.21) **Zeros OR: 1.14 (1.01, 1.28)**
CD3^+^CD8^+^ naive	1.07 (0.97; 1.19)	**0.95 (0.93, 0.96)**	0.95 (0.81, 1.11)	1.03 (0.91, 1.17)	1.05 (0.98, 1.12)	1.03 (0.91, 1.16)	Count IRR: 0.99 (0.82, 1.20) Zeros OR: 1.10 (0.97, 1.25)
CD3^+^CD8^+^ regulatory	1.02 (0.92; 1.14)	**0.93 (0.92, 0.94)**	0.93 (0.81, 1.06)	0.98 (0.88, 1.1)	1.02 (0.96, 1.09)	0.98 (0.88, 1.10)	Count IRR: 0.96 (0.82, 1.13) Zeros OR: 1.05 (0.93, 1.19)
**Low percent zeros**							
CD3^+^CD4^+^ memory	1.04 (0.94; 1.15)	**0.88 (0.86, 0.89)**	**0.88 (0.79, 0.97)**	**0.88 (0.81, 0.96)**	1.01 (0.94, 1.07)	**0.88 (0.81, 0.96)**	**Count IRR: 0.84 (0.76, 0.93) Zeros OR: 1.19 (1.03, 1.36)**
CD3^+^CD8^+^ memory	0.95 (0.86; 1.05)	**0.95 (0.94, 0.97)**	0.95 (0.84, 1.08)	0.97 (0.88, 1.07)	0.98 (0.92, 1.05)	0.97 (0.88, 1.07)	Count IRR: 0.94 (0.81, 1.08) Zeros OR: 1.09 (0.95, 1.24)
CD3^-^ immune cells	1.08 (0.97; 1.20)	**1.05 (1.04, 1.06)**	1.05 (0.99, 1.11)	1.04 (0.99, 1.09)	1.03 (0.99, 1.07)	-	

Abbreviations: Inf, Infinity, unstable high value; IRR, incidence rate ratio; NA, non-converging value; NB, negative binomial; OFCCR, Ontario Family Colon Cancer Registry;. OR, odds ratio; ZINB, zero-inflated negative binomial.

Adjusted for age, sex, study batch (Harvard/OFCCR), and cancer site (proximal, distal, rectal, and missing).

Bold values indicate *P*-value <0.05.

Cells with dashed lines indicate cases where models were not run because they were unsuitable based on the percentage of zeros (eg, zero-inflated models were skipped for CD3^–^ immune cells, and count models were avoided for subsets with a high percentage of zeros).

Ordinal logistic regression results were previously published in Wesselink et al.^22^

a The binary component of the ZINB model only included tissue area as a covariate, and did not model our exposure of interest due to model instability.

### Type I error

**Figure 2:**
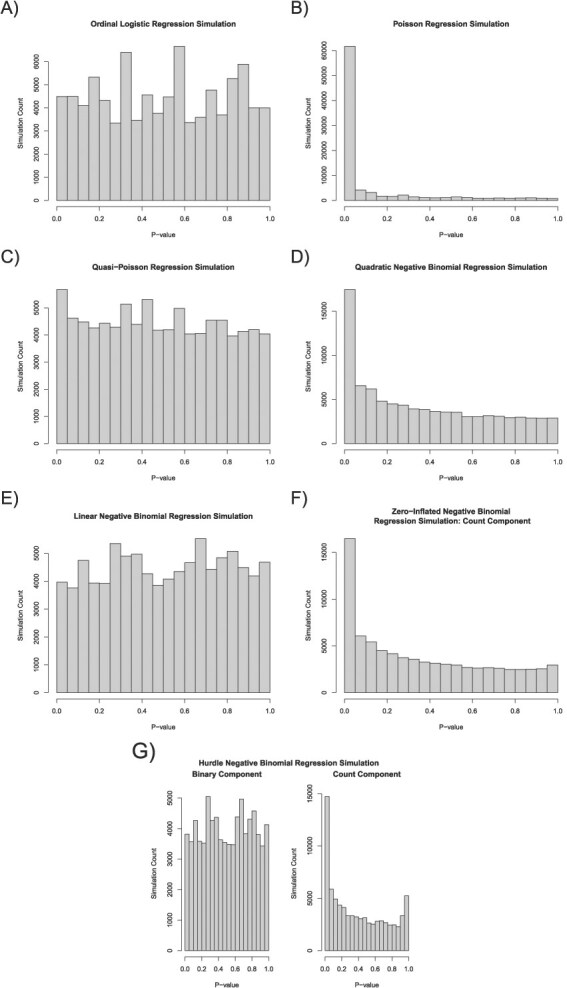
Type I error real-world data simulation study results for (A) ordinal logistic regression, (B) Poisson regression, (C) quasi-Poisson regression, (D) quadratic NB regression, (E) linear NB regression, (F) ZINB regression, (G) hurdle negative binomial regression. A toal of 10 000 simulations per epithelial tissue area immune cell subset (9 subsets total) = 90 000 tests. ZINB and hurdle models were run for all subsets except CD3^–^ immune cells, due to very low zero counts in that subset (80 000 tests total). Less than 1% of *P*-values did not converge for the Poisson, quasi-Poisson, negative binomial, and the count component of the hurdle negative binomial models, and all the nonconverging models were for the double negative naive subset. The binary component of the hurdle negative binomial model had no convergence issues. The binary component of the ZINB model, which did not include the exposure of interest and only the area term as a predictor, did not converge 45% of the time, while the count component which did model a simulation of the exposure of interest did not converge <1% of the time. Abbreviations: NB, negative binomial; ZINB, zero-inflated negative binomial.

Figure 2 shows the histograms of *P*-value distributions of ordinal logistic, Poisson, quasi-Poisson, quadratic NB, linear NB, ZINB, and hurdle models under the null distribution using our real data-based simulated datasets in which the exposure of interest was randomly permuted. Both the ordinal logistic and linear NB regression yielded approximately uniform *P*-value distributions, indicating well-controlled type I error (Figure 2A and E). However, the Poisson and quadratic NB models exhibited an excess of low *P*-values, suggesting inflation of false positives (Figure 2B and D). The quasi-Poisson model showed a largely uniform distribution, with a modest excess of low *P*-values (Figure 2C).

When stratified by high, medium, and low percentages of zeros, the quadratic NB model exhibited an excess of small *P*-values across all strata (Figure S1A-C). After excluding influential observations identified using Cook’s distance,^43^ the resulting *P*-value distributions continued to indicate inflated false-positive rates (Figure S1D). The Poisson model likewise showed an excess of small *P*-values across all strata (Figure S2). For the quasi-Poisson model, we observed a modest excess of small *P*-values across all strata. In addition, the *P*-value distribution was noticeably irregular for high percentage of zeros, suggesting instability in this setting (Figure S3). Both the ZINB model and hurdle models showed an excessive level of low *P*-values in the count component (Figure 2F and G). The *P*-value distribution for the binary component of the hurdle model was fairly uniform (Figure 2G). When stratified by high, medium, and low percentages of zeros, the count component of both the ZINB and hurdle models showed an excess of small *P*-values across strata (Figure S4A-C). In settings with a high percentage of zeros, the ZINB model also produced an excess of *P*-values near 1.

When fitting the ZINB model, we observed an overall nonconvergence rate of 45%, with 45% failing in the binary component and less than 1% failing in the count component. Because the zero-inflation and count components are jointly estimated, failure of either component to converge invalidates the parameter estimates of the entire model. For the Poisson, quasi-Poisson, quadratic NB, and the count component of the hurdle NB models, fewer than 1% of models failed to converge; all nonconverging cases were for the double negative naive subset, which had the highest percentage of zeros (96%). No convergence issues were observed for the linear NB and the binary component of the hurdle NB model.

### Power

**Figure 3:**
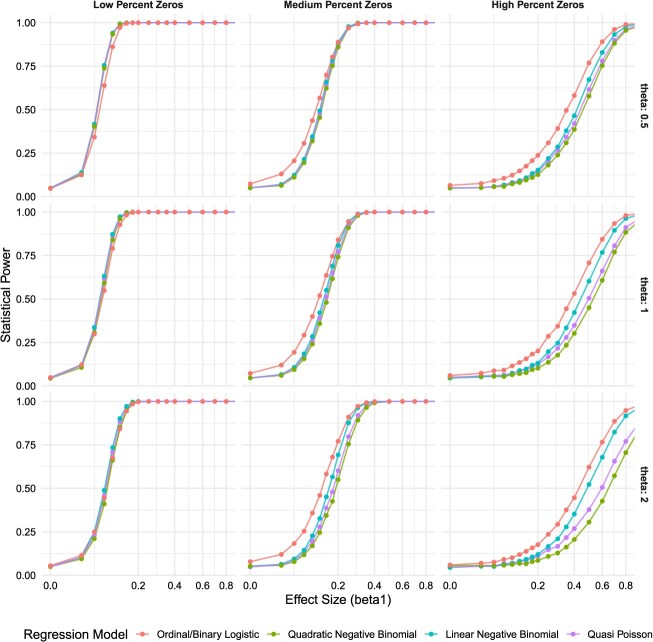
Power simulation across different conditions and statistical models under the linear negative binomial (NB) model.

Figure 3 shows the statistical power of the ordinal logistic, quasi-Poisson, quadratic NB, and linear NB as a function of the regression coefficient for the association between the exposure of interest and the outcome where the data was simulated under linear NB distribution. Results under the quadratic NB distribution are shown in Figure S5. Results are shown across scenarios defined by the percentage of zeros (low, medium, and high; columns) and the dispersion level (*θ* = 0.5, 1, 2; rows).

Under the linear NB distribution, for scenarios with low percentages of zeros, all models exhibited broadly similar power across *θ* values (Figure 3). For low and medium percentages of zeros, ordinal logistic regression had the highest power in all dispersion settings, followed by the linear NB model with the second-highest power. When the data were simulated under a quadratic NB distribution, for scenarios with a high percentage of zeros, all models exhibited broadly similar power across *θ* values, with ordinal logistic regression having the highest power, although the differences among models were modest (Figure S5). Likewise, under overdispersed settings (*θ* = 2), power was comparable across all models regardless of the percentage of zeros. In contrast, when *θ* was 1 or 0.5 and the percentage of zeros was low or medium, the quasi-Poisson model consistently achieved the highest power, followed by the NB and ordinal logistic models, with the linear NB having the lowest power.

Notably, although the data were generated under multiple NB models, fitting an NB model did not necessarily yield the highest power. Under the linear NB distribution, the greater power of the ordinal logistic model came at the cost of a slight inflation of type I error (up to 8%) (Table S2). Under the quadratic NB distribution, the quasi-Poisson and linear NB model showed modest type I error inflation, yet consistently demonstrated the lowest power.

Histograms of simulated count distributions under the linear NB model for representative examples with high, medium, and low percentages of zeros across different dispersion settings are shown in Figures S6A, S6B, and S6C.

### Revisit model comparisons after simulation

We now revisited the results from the different modeling approaches for MSI status and calcium intake. For the strong exposure, MSI status, conclusions were generally consistent across the models; however, the magnitude of the *P*-values mirrored the simulation findings (Tables 2 and S3). Specifically, *P*-values tended to be smaller for the Poisson and quadratic NB models and larger for the quasi-Poisson, ordinal logistic regression, and linear NB models. Consistent with this pattern, MSI status was identified as being statistically significantly associated with higher levels of the CD3^+^CD4^+^ memory T cells using the quadratic NB model (IRR = 1.59; 95% CI, 1.25-2.05) when the ordinal logistic regression result was not statistically significant (OR = 1.28; 95% CI, 0.96-1.71), nor the linear NB model. These discrepancies align with the potentially inflated false-positive rates observed for the quadratic NB model in the simulation study.

For the weak exposure, total calcium intake, results and *P*-values varied more substantially across modeling approaches (Tables 3 and S4). Now knowing the high likelihood of false positives observed for the Poisson model, statistically significant associations identified by Poisson regression for several immune subsets, which were not supported by the quasi-Poisson (except for CD3^+^CD4^+^ memory T cells) or ordinal logistic regression, are likely to represent false positives. For CD3^+^CD4^+^ memory T cells, results from the Poisson, quasi-Poisson, quadratic NB, ZINB, and hurdle count model were statistically significant, whereas those from the ordinal logistic and linear NB results were borderline. These findings provide modest statistical evidence for this association.

## Discussion

We evaluated statistical models for T cell subset densities in CRC tumors, using a strong exposure (MSI) and a weak exposure (calcium intake) as contrasting examples. For the strong exposure, results between models were consistent in direction; however, for the weak exposure, greater differences between models were observed. Hurdle NB models exhibited better stability than ZINB. ZINB’s dual-component origin for zeros creates identifiability challenges, whereas hurdle models simplify estimation by treating zeros as a single process. Although results may point in the same direction and be consistent for different approaches, particularly for strong exposures, model assumptions and pitfalls should be carefully checked.

Simulations revealed excessive false positives for Poisson, quadratic NB, the count components of the hurdle and ZINB models, and a modest inflation of false positives for the quasi-Poisson. Although results from the Poisson and quadratic NB models were generally directionally consistent with other modeling approaches, at least for strong exposures, such consistency does not guarantee valid inference. Given the inflated false-positive rates associated with the Poisson and quadratic NB approach, it is difficult to determine if a statistically significant result reflects a true association attributable to increased power from modeling the continuous counts outcome (as opposed to categorization in the ordinal logistic regression) or instead arises from model misspecification and type I error inflation. While the quasi-Poisson model still had some excess of small *P*-values, the inflation is modest, and it has better power than other approaches for low and medium percentage of zero counts and may therefore be worth considering. The linear NB model also performed well, having a relatively uniform distribution of *P*-values under the null and comparable power to ordinal logistic regression in simulations under the linear NB distribution, although it still underperformed the power of the ordinal logistic regression model. Ordinal logistic regression, on the contrary, emerges as a robust approach with competitive power performance across all scenarios while maintaining reasonable control of type I error. Notably, the findings of this study are likely transferable to a broader array of assays beyond mIF data as multiomic technologies become more widely adopted, including microbiome abundance and spatial transcriptional profiling data. The challenges posed by high percentages of zeros and/or over dispersed data identified in this study are increasingly common in molecular epidemiologic studies and warrant careful consideration and further investigation.

Comparing ordinal logistic regression with count-based models reveals distinct advantages and limitations regarding data granularity and interpretation. The ordinal logistic regression model offers the advantage of robustness; it handles extreme values and excess zeros well without complex distribution assumptions, provided the proportional odds assumption holds and categorization is appropriate for the percentage of zeros. However, its interpretation is abstract: effect estimates are expressed as the odds of belonging to a higher quantile rather than a direct quantity of cells. Additionally, the necessary categorization of continuous outcomes can be arbitrary and may reduce statistical power. In contrast, count-based models, such as quasi-Poisson and linear NB regression, retain the continuous nature of the data and handle overdispersion by allowing variance to differ from the mean. The interpretation of these count models is arguably more intuitive for biological applications, as exponentiated coefficients represent IRRs that describe a direct multiplicative change (or fold-difference) in cell density. While full probability models like the linear NB model are more sensitive to distributional assumptions than ordinal logistic regression, they allow for the prediction of actual counts and avoid the information loss inherent in categorization.

Our study has many strengths. We utilized 7 different models to identify how associations differed for both a strong and weak exposure in a large, population-based mIF study. Our mIF panel allows for generation of function-specific T cell densities with differing percentage of zero counts, allowing us to assess the type I error for different distributions of zeros. Our integrative CRC tumor databases^23^^,^^24^ allowed us to examine different exposures including clinical, demographic, and epidemiological variables. We also conducted 2 simulation studies based on real data to assess type I error and power. Several alternative approaches warrant future investigation. We focused on absolute densities, excluding relative abundance models like beta-binomial.^11^ We also averaged multiple TMAs cores per subject to reduce the complexity and dimensionality of our dataset as we have published previously^21^^,^^40^; however, other groups have explored random-effect term models accounting for repeated measures per subject,^44^ which may be a relevant approach to consider.^11^

In conclusion, we recommend considering ordinal logistic regression, linear NB, and quasi-Poisson models for the analysis of overdispersed and zero-inflated data. It is important to examine the adequacy of models when dealing with nonstandard data, here, excessive zero counts and overdispersion, to ensure accurate and reliable inference for cutting-edge omics data.

## Supplementary Material

Web_Material_kwag127

## Data Availability

The data underlying this article will be shared on reasonable request to the corresponding authors.
