# Supplementary material for: Evaluating statistical models for overdispersed multiomics data: a multiplex immunofluorescence case study
Source: Am J Epidemiol. 2026 Jun 15;195(8):2307–18. doi: 10.1093/aje/kwag127 (PMC13437073; doi:10.1093/aje/kwag127)
Supplement: Supplementary file 1 [file web_material_kwag127.docx]

**Supplemental Materials: Evaluating statistical models for overdispersed multi-omics data: A multiplex immunofluorescence case study**

Claire E. Thomas^1^*, Evertine Wesselink^2^*, Yasutoshi Takashima^3^, Jeroen R. Huyghe^1^, Daniel D. Buchanan^4,5,6^, Robert C. Grant^7^, Andressa Dias Costa^8^, Tomotaka Ugai^9,10^, Shuji Ogino^9,10,11,12^, Jonathan A. Nowak^9,13^, Ulrike Peters^1,14^, Amanda I. Phipps^1,14^, Li Hsu^1,15^

^1^Public Health Sciences Division, Fred Hutchinson Cancer Center, Seattle, Washington, USA., ^2^Division of Molecular Pathology, Netherlands Cancer Institute-Antoni van Leeuwenhoek Hospital, Amsterdam, The Netherlands., ^3^Department of Medical Oncology, Dana-Farber Cancer Institute, Boston, MA, USA., ^4^Colorectal Oncogenomics Group, Department of Clinical Pathology, Melbourne Medical School, The University of Melbourne, Parkville, Australia., ^5^Collaborative Centre for Genomic Cancer Medicine, The University of Melbourne, Victorian Comprehensive Cancer Centre, Parkville, Australia., ^6^Genomic Medicine and Family Cancer Clinic, The Royal Melbourne Hospital, Parkville, Victoria, Australia., ^7^Division of Medical Oncology and Hematology, Princess Margaret Cancer Centre, University Health Network, Toronto, Ontario, Canada, ^8^Department of Medical Oncology, Dana-Farber Cancer Institute and Harvard Medical School, Boston, Massachusetts, USA, ^9^Program in MPE Molecular Pathological Epidemiology, Department of Pathology, Brigham and Women’s Hospital and Harvard Medical School, Boston, Massachusetts, USA., ^10^Department of Epidemiology, Harvard T.H. Chan School of Public Health, Boston, Massachusetts, USA., ^11^Broad Institute of MIT and Harvard, Cambridge, Massachusetts, USA., ^12^Institute of Science Tokyo, Tokyo, Japan, ^13^Department of Oncologic Pathology, Dana-Farber Cancer Institute, Boston, Massachusetts, USA., ^14^Department of Epidemiology, University of Washington, Seattle, Washington, USA., ^15^Department of Biostatistics, University of Washington, Seattle, Washington, USA.

Co-first and co-corresponding authors

**Table of Contents**

- **Supplemental Tables**
  - Table S1: Overview strengths and limitations as well as interpretation of modeling approaches examined for varying levels of overdispersed zero-inflated count and density multiplex immunofluorescence data
  - Table S2: Type 1 error for simulated data from power simulation study under the type I & linear NB distributions (β=0)
  - Table S3: Association between microsatellite instability status (strong exposure) and T cell subset densities utilizing multiple modeling approaches (N=1,235), including p-values for significance comparison
  - Table S4: Association between total calcium intake (weak exposure) and T cell subset densities utilizing multiple modeling approaches (N=936), including p-values for significance comparison
- **Supplemental Figures**
  - Figure S1: Type I Error real-world data simulation results for quadratic negative binomial models stratified by percentage of zero counts: A) high, B) medium, C) low, and D) medium and low zeros combined, removing influential points with high Cook’s Distance values
  - Figure S2: Type I Error real-world data simulation results for Poisson models stratified by percentage of zero counts: A) high, B) medium, C) low
  - Figure S3: Type I Error real-world data simulation results for quasi-Poisson models stratified by percentage of zero counts: A) high, B) medium, C) low
  - Figure S4: Type I Error real data based simulation results for the count component of ZINB & hurdle negative binomial models stratified by percentage of zero counts: A) high, B) medium, C) low
  - Figure S5: Power simulation across different conditions & statistical models under a quadratic NB distribution
  - Figure S6A: Distribution of simulated counts for a high percentage zeros subset under a linear NB distribution
  - Figure S6B: Distribution of simulated counts for a medium percentage zeros subset under a linear NB distribution
  - Figure S6C: Distribution of simulated counts for a low percentage zeros subset under a linear NB distribution

**Table S1: Overview strengths and limitations as well as interpretation of modeling approaches examined for varying levels of overdispersed zero-inflated count and density multiplex immunofluorescence data**

| Statistical method | Strengths | Limitations | Interpretation |
| --- | --- | --- | --- |
| Ordinal logistic regression | Simple and computationally stable  Robust to extreme values and excess zeros  Intuitive when relative ranking is of interest | Requires categorization of continuous outcomes, which can be arbitrary due to difficulty in choosing clinically meaningful cut-points    May reduce power   Relies on the proportional odds assumption (i.e., constant effect across adjacent categories) | Exponentiated coefficients represent the multiplicative change in the odds of being in a higher outcome quantile category for a one-unit increase in the exposure. Exponentiating β gives the odds ratio (OR). Example: MSI-high tumors had 1.65 times the odds of higher quantile of epithelial CD3⁺CD4⁺ naïve T-cell density compared with MSI-low/MSS tumors. |
| Poisson count model | Natural baseline model for count data  Parameters directly describe multiplicative changes in expected counts (IRRs)  Easily extended to rates (offsets), mixed models (random effects), or nonlinear effects (splines) | Assumes equality of mean and variance, often violated in real data, potentially leading to invalid inference  Sensitive to excess zeros | Exponentiated coefficients represent the multiplicative change in the expected count for a one-unit increase in the exposure. Exponentiating β gives the incidence rate ratio (IRR).  Example: MSI-high tumors had 1.2 times the density of epithelial CD3⁺CD4⁺ naïve T-cells compared with MSI-low/MSS tumors. |
| Quasi-Poisson count model | Extension of Poisson regression allowing variance to differ from the mean | Limited handling of high percentages of zeros | Exponentiated coefficients represent the multiplicative change in the expected count for a one-unit increase in the exposure. Exponentiating β gives the incidence rate ratio (IRR).  Example: MSI-high tumors had 1.2 times the density of epithelial CD3⁺CD4⁺ naïve T-cells compared with MSI-low/MSS tumors. |
| Quadratic Negative binomial (NB) count model | Retains continuous count information    Models overdispersion via a quadratic mean-variance relationship | Does not explicitly account for high percentages of zeros  Misspecification may still lead to biased inference | Exponentiated coefficients represent the multiplicative change in the expected count for a one-unit increase in the exposure. Exponentiating β gives the incidence rate ratio (IRR).  Example: MSI-high tumors had 1.7 times the density of epithelial CD3⁺CD4⁺ naïve T-cells compared with MSI-low/MSS tumors. |
| Linear negative binomial (NB) count model | Retains continuous count information    Models overdispersion via a linear mean-variance relationship | Does not explicitly account for high percentages of zeros | Exponentiated coefficients represent the multiplicative change in the expected count for a one-unit increase in the exposure. Exponentiating β gives the incidence rate ratio (IRR).    Example: MSI-high tumors had 1.4 times the density of epithelial CD3⁺CD4⁺ naïve T-cells compared with MSI-low/MSS tumors. |
| Zero-inflated negative binomial (ZINB) model | Simultaneously models overdispersion and excess zeros    Assumes that zeros arise from two sources: *structural zeros*, representing subjects not at risk for the outcome, and *sampling zeros*, representing at-risk subjects who happen to have zero counts. | Interpretation more complex (two different model components that should be interpreted together)    Identifiability can be challenging, particularly with limited sample sizes | *Count component:* Exponentiated coefficients represent the multiplicative change in the expected count for a one-unit increase in the exposure, conditional on the subject being at risk (i.e. not a structural zero). Exponentiating β of the count component gives the incidence rate ratio (IRR).  Example: MSI-high tumors had 5 times higher expected counts of epithelial double negative memory T cells compared with MSI-low/MSS tumors, among the non-structural-zero population.  *Zero-inflation component:* Exponentiated coefficients describe the odds of belonging to the always-zero group. Exponentiating β of the binary component gives the odds ratio (OR).  Example: MSI-high tumors had lower odds of being structurally zero compared with MSI-low/MSS tumors. |
| Hurdle negative binomial model | Separates zero vs non-zero outcomes, allowing distinct biological interpretation of presence and abundance.    Flexible handling of excess zeros and overdispersion | Assumes all zeros arise from a single process (no latent always-zero group)    Requires sufficient non-zero observations for stable estimation | *Positive count component:* Exponentiated coefficients represent the multiplicative change in the expected count for a one-unit increase in the exposure, conditional on the outcome being non-zero. Exponentiating β of the count component gives the incidence rate ratio (IRR).  Example: MSI-high tumors had 7 times higher expected counts of epithelial double negative memory T cells compared with MSI-low/MSS tumors, among those with epithelial double negative memory T cells present.  *Zero component:* Exponentiated coefficients represent the likelihood of positivity in your data, or the multiplicative change in the odds of being positive for a one-unit increase in the exposure variable. Exponentiating β of the binary component gives the odds ratio (OR).  Example: MSI-high tumors were 2.9 times as likely to be positive for epithelial double negative memory T cells compared with MSI-low/MSS tumors. |

**Table S2: Type 1 error for simulated data from power simulation study under the type I & linear NB distributions (β=0)**

| Simulated Distribution | Percentage of zeros | Dispersion | β | Quasi-Poisson | Linear NB | Quadratic NB | Ordinal logistic | N Convergence |
| --- | --- | --- | --- | --- | --- | --- | --- | --- |
| Linear NB | Low | 0.5 | 0 | 0.05 | 0.05 | 0.05 | 0.05 | 10,000 |
|  | Low | 1 | 0 | 0.05 | 0.05 | 0.04 | 0.05 | 10,000 |
|  | Low | 2 | 0 | 0.05 | 0.05 | 0.05 | 0.05 | 10,000 |
|  | Medium | 0.5 | 0 | 0.05 | 0.05 | 0.05 | 0.07 | 10,000 |
|  | Medium | 1 | 0 | 0.05 | 0.05 | 0.05 | 0.07 | 10,000 |
|  | Medium | 2 | 0 | 0.05 | 0.05 | 0.05 | 0.08 | 10,000 |
|  | High | 0.5 | 0 | 0.05 | 0.05 | 0.05 | 0.06 | 10,000 |
|  | High | 1 | 0 | 0.05 | 0.05 | 0.05 | 0.06 | 10,000 |
|  | High | 2 | 0 | 0.05 | 0.04 | 0.05 | 0.06 | 10,000 |
| Quadratic NB | Low | 0.5 | 0 | 0.08 | 0.08 | 0.05 | 0.05 | 10,000 |
|  | Low | 1 | 0 | 0.08 | 0.08 | 0.06 | 0.05 | 10,000 |
|  | Low | 2 | 0 | 0.07 | 0.07 | 0.05 | 0.05 | 10,000 |
|  | Medium | 0.5 | 0 | 0.07 | 0.06 | 0.05 | 0.06 | 10,000 |
|  | Medium | 1 | 0 | 0.07 | 0.06 | 0.05 | 0.06 | 10,000 |
|  | Medium | 2 | 0 | 0.06 | 0.05 | 0.05 | 0.07 | 10,000 |
|  | High | 0.5 | 0 | 0.05 | 0.05 | 0.05 | 0.06 | 9,999 |
|  | High | 1 | 0 | 0.05 | 0.06 | 0.05 | 0.06 | 9,978 |
|  | High | 2 | 0 | 0.05 | 0.09 | 0.05 | 0.07 | 9,929 |

**Table S3: Association between microsatellite instability status (strong exposure) and T cell subset densities utilizing multiple modeling approaches (N=1,235), including p-values for significance comparison**

| Epithelial Tissue Area Immune Subset Density | Ordinal Logistic, densities:  OR (95% CI)  P-value | Poisson, count: IRR (95% CI)  P-value | Quasi-Poisson, count: IRR (95% CI)  P-value | Quadratic NB, count: IRR (95% CI)  P-value | Linear NB, count:  IRR (95% CI)  P-value | Zero-inflated NB, count: IRR for count*:  IRR (95% CI)  P-value | Hurdle NB, count: OR for binary and IRR for count  IRR or OR (95% CI)  P-value |
| --- | --- | --- | --- | --- | --- | --- | --- |
| **High Percent Zeros** |  |  |  |  |  |  |  |
| Double negative naive | **2.41 (1.16, 4.89)**  **P = 0.016** | - | - | - | - | 4.17 (NA, NA)  P = NA | Count IRR: 19.28 (0, Inf)  P = 0.931  Zeros OR: 3.55 (0.97, 12.97)  P = 0.055 |
| Double negative memory | **2.49 (1.68, 3.70)**  **P = 5.4E-06** | **-** | - | - | **-** | 4.92 (NA, NA)  P = NA | **Count IRR: 7.27 (2.21, 23.96)**  **P = 0.001**  **Zeros OR: 2.89 (1.75, 4.78)**  **P = 3.5E-05** |
| **Medium Percent Zeros** |  |  |  |  |  |  |  |
| CD3^+^CD4^+^ naive | **1.65 (1.22, 2.22)**  **P = 0.001** | **1.22 (1.13, 1.31)**  **P = 1.9E-07** | 1.22 (0.78, 1.87)  P = 0.364 | **1.65 (1.17, 2.35)**  **P = 0.004** | **1.35 (1.08, 1.68)**  **P = 0.009** | **1.61 (1.14, 2.29)**  **P = 0.007** | **Count IRR: 1.81 (1.05, 3.09)**  **P = 0.031**  Zeros OR: 1.37 (0.97, 1.92)  P = 0.071 |
| CD3^+^CD4^+^ regulatory | **2.03 (1.51, 2.74)**  **P = 3.5E-06** | **1.63 (1.48, 1.8)**  **9.8E-23** | **1.63 (1.13, 2.34)**  **P = 0.008** | **1.83 (1.34, 2.53)**  **P = 1.3E-04** | **1.62 (1.31, 2.00)**  **P = 9.3E-06** | **1.80 (1.31, 2.47)**  **P = 2.8E-04** | **Count IRR: 1.92 (1.21, 3.05)**  **P = 0.005**  **Zeros OR: 1.82 (1.31, 2.54)**  **P = 3.8E-04** |
| CD3^+^CD8^+^ naive | **3.49 (2.57, 4.75)**  **P = 1.2E-15** | **6.16 (5.95, 6.37)**  **P < 1E-100** | **6.16 (4.64, 8.20)**  **P = 2.7E-34** | **4.76 (3.59, 6.40)**  **P = 4.9E-25** | **2.27 (1.91, 2.70)**  **P = 1.5E-19** | **4.69 (3.51, 6.25)**  **P = 8.0E-26** | **Count IRR: 4.35 (2.96, 6.39)**  **P = 6.4E-14**  **Zeros OR: 3.84 (2.56, 5.76)**  **P = 9.0E-11** |
| CD3^+^CD8^+^ regulatory | **3.04 (2.24, 4.13)**  **P = 1.2E-12** | **5.24 (5.06, 5.43)**  **P < 1E-100** | **5.24 (4.07, 6.77)**  **P = 2.7E-35** | **4.66 (3.53, 6.24)**  **P = 4.8E-26** | **2.12 (1.79, 2.52)**  **P = 1.9E-17** | **4.60 (3.47, 6.11)**  **P = 3.9E-26** | **Count IRR: 4.49 (3.15, 6.41)**  **P = 1.2E-16**  **Zeros OR: 3.11 (2.06, 4.69)**  **P = 5.9E-08** |
| **Low Percent Zeros** |  |  |  |  |  |  |  |
| CD3^+^CD4^+^ memory | 1.28 (0.96, 1.71)  P = 0.093 | **1.53 (1.46, 1.61)**  **P = 8.6E-68** | **1.53 (1.19, 1.96)**  **P = 8.7E-04** | **1.59 (1.25, 2.05)**  **P = 1.3E-04** | 1.14 (0.96, 1.34)  P = 0.128 | **1.60 (1.25, 2.05)**  **2.1E-04** | **Count IRR: 1.86 (1.39, 2.49)**  **P = 2.7E-05**  Zeros OR: 0.92 (0.63, 1.34)  P = 0.659 |
| CD3^+^CD8^+^ memory | **2.82 (2.10, 3.78)**  **P = 4.4E-12** | **3.23 (3.1, 3.36)**  **P < 1E-100** | **3.23 (2.48, 4.21)**  **P = 1.3E-17** | **3.63 (2.79, 4.75)**  **4.5E-22** | **1.82 (1.55, 2.14)**  **P = 6.7E-13** | **3.63 (2.78, 4.73)**  **P = 1.8E-21** | **Count IRR: 3.78 (2.64, 5.42)**  **P = 4.3E-13**  **Zeros OR: 2.76 (1.78, 4.27)**  **P = 5.2E-06** |
| CD3^-^ immune cells | **2.10 (1.58, 2.79)**  **P = 3.0E-07** | **1.59 (1.57, 1.62)**  **P < 1E-100** | **1.59 (1.39, 1.82)**  **P = 2.0E-11** | **1.55 (1.35, 1.78)**  **4.3E-10** | **1.42 (1.28, 1.57)**  **P = 2.9E-11** | - | - |

Adjusted for age, sex, study batch (Harvard/OFCCR), and cancer site (proximal, distal, rectal, missing).

**Bolded:** P-value < 0.05, Inf = Infinity, unstable high value, NA= non-converging value, NB = negative binomial

Cells with dashed lines indicate cases where models were not run because they were unsuitable based on the percentage of zeros (e.g., zero-inflated models were skipped for CD3⁻ immune cells, and count models were avoided for subsets with a high percentage of zeros).

Ordinal logistic regression results were previously published in Frontiers in Immunology, <https://doi.org/10.3389/fimmu.2024.1505896>

*The binary component of the ZINB model only included tissue area as a covariate, and did not model our exposure of interest due to model instability.

**Table S4: Association between total calcium intake (weak exposure) and T cell subset densities utilizing multiple modeling approaches (N=936), including p-values for significance comparison**

| Epithelial Tissue Area Immune Subset Density | Ordinal Logistic, densities:  OR (95% CI)  P-value | Poisson, count: IRR (95% CI)  P-value | Quasi-Poisson, count: IRR (95% CI)  P-value | Quadratic NB, count: IRR (95% CI) | Linear NB, count:  IRR (95% CI) | Zero-inflated NB, count: IRR for count*:  IRR (95% CI)  P-value | Hurdle NB, count: OR for binary and IRR for count  IRR or OR (95% CI)  P-value |
| --- | --- | --- | --- | --- | --- | --- | --- |
| **High Percent Zeros** |  |  |  |  |  |  |  |
| Double negative naive | 1.25 (0.92; 1.71)  P = 0.081 | - | - | - | **-** | 1.62 (0.84, 3.12)  P = 0.148 | Count IRR: 1.95 (0.01, 395.83) P = 0.806  Zeros OR: 1.44 (0.86, 2.42)  P = 0.169 |
| Double negative memory | 1.03 (0.87; 1.21)  P = 0.581 | - | - | - | - | 1.02 (0.81, 1.30)  P = 0.842 | Count IRR: 0.8 (0.52, 1.22)  P = 0.300  Zeros OR: 1.08 (0.87, 1.34)  P = 0.503 |
| **Medium Percent Zeros** |  |  |  |  |  |  |  |
| CD3^+^CD4^+^ naive | 1.01 (0.90; 1.12)  P = 0.906 | **1.14 (1.11, 1.17)**  **P = 2.5E-20** | 1.14 (0.96, 1.35)  P = 0.148 | 1.12 (0.99, 1.28)  P = 0.073 | 1.01 (0.93, 1.10)  P = 0.803 | 1.13 (0.99, 1.28)  P = 0.074 | **Count IRR: 1.30 (1.07, 1.58)**  **P = 0.009**  Zeros OR: 0.98 (0.86, 1.10)  P = 0.705 |
| CD3^+^CD4^+^ regulatory | **1.14 (1.03; 1.27)**  **P = 0.014** | **1.16 (1.12, 1.21)**  **P = 9.2E-14** | 1.16 (1, 1.35)  P = 0.056 | 1.09 (0.97, 1.23)  P = 0.126 | 1.09 (1.00, 1.18)  P = 0.052 | 1.09 (0.97, 1.23)  P = 0.157 | Count IRR: 1.02 (0.86, 1.21)  P = 0.803  **Zeros OR: 1.14 (1.01, 1.28)**  **P = 0.038** |
| CD3^+^CD8^+^ naive | 1.07 (0.97; 1.19)  P = 0.184 | **0.95 (0.93, 0.96)**  **P = 2.0E-15** | 0.95 (0.81, 1.11)  P = 0.500 | 1.03 (0.91, 1.17)  P = 0.595 | 1.05 (0.98, 1.12)  P = 0.205 | 1.03 (0.91, 1.16)  P = 0.655 | Count IRR: 0.99 (0.82, 1.20)  P = 0.939  Zeros OR: 1.10 (0.97, 1.25)  P = 0.146 |
| CD3^+^CD8^+^ regulatory | 1.02 (0.92; 1.14)  P = 0.689 | **0.93 (0.92, 0.94)**  **P = 3.5E-22** | 0.93 (0.81, 1.06)  P = 0.295 | 0.98 (0.88, 1.10)  P = 0.767 | 1.02 (0.96, 1.09)  P = 0.521 | 0.98 (0.88, 1.10)  P = 0.738 | Count IRR: 0.96 (0.82, 1.13)  P = 0.629  Zeros OR: 1.05 (0.93, 1.19)  P = 0.433 |
| **Low Percent Zeros** |  |  |  |  |  |  |  |
| CD3^+^CD4^+^ memory | 1.04 (0.94; 1.15)  P = 0.485 | **0.88 (0.86, 0.89)**  **P = 1.1E-38** | **0.88 (0.79, 0.97)**  **P = 0.010** | **0.88 (0.81, 0.96)**  **P = 0.004** | 1.01 (0.94, 1.07)  P = 0.875 | **0.88 (0.81, 0.96)**  **P = 0.003** | **Count IRR: 0.84 (0.76, 0.93)**  **P = 3.9E-04**  **Zeros OR: 1.19 (1.03, 1.36)**  **P = 0.016** |
| CD3^+^CD8^+^ memory | 0.95 (0.86; 1.05)  P = 0.321 | **0.95 (0.94, 0.97)**  **P = 8.5E-08** | 0.95 (0.84, 1.08)  P = 0.462 | 0.97 (0.88, 1.07)  P = 0.578 | 0.98 (0.92, 1.05)  P = 0.550 | 0.97 (0.88, 1.07)  P = 0.570 | Count IRR: 0.94 (0.81, 1.08)  P = 0.372  Zeros OR: 1.09 (0.95, 1.24)  P = 0.214 |
| CD3^-^ immune cells | 1.08 (0.97; 1.20)  P = 0.141 | **1.05 (1.04, 1.06)**  **8.9E-46** | 1.05 (0.99, 1.11)  P = 0.109 | 1.04 (0.99, 1.09)  P = 0.158 | 1.03 (0.99, 1.07)  P = 0.197 | - | - |

Adjusted for age, sex, study batch (Harvard/OFCCR), and cancer site (proximal, distal, rectal, missing).

**Bolded:** P-value < 0.05, Inf = Infinity, unstable high value, NA= non-converging value, NB= negative binomial.

Cells with dashed lines indicate cases where models were not run because they were unsuitable based on the percentage of zeros (e.g., zero-inflated models were skipped for CD3⁻ immune cells, and count models were avoided for subsets with a high percentage of zeros).

Ordinal logistic regression results were previously published in Cancer Prevention Research, <https://doi.org/10.1158/1940-6207.CAPR-25-0023>

*The binary component of the ZINB model only included tissue area as a covariate, and did not model our exposure of interest due to model instability.

**Figure S1: Type I Error real-world data simulation results for quadratic negative binomial models stratified by percentage of zero counts: A) high, B) medium, C) low, and D) medium and low zeros combined, removing influential points with high Cook’s Distance values**

A)

B)

C)

D) Medium and low zeros combined, removing influential points with high Cook’s Distance values

Footnote: High, medium, and low include 20,000, 40,000, and 30,000 simulation counts, respectively.

To evaluate if excessive false positives were driven by influential observations, we conducted an additional simulation analysis for the NB model after removing influential points (Supplemental Figure 1D). Influential points were identified by Cook’s distance,^43^ where any observation with a value greater than three times the mean Cook's distance was deemed influential and removed. This additional analysis was limited to T cell subsets with low and medium levels of zeros because model fitting became highly unstable after influential point removal in subsets with high percentage of zero counts.

**Figure S2: Type I Error real-world data simulation results for Poisson models stratified by percentage of zero counts: A) high, B) medium, C) low**

A)

B)

C)

Footnote: High, medium, and low include 20,000, 40,000, and 30,000 simulation counts, respectively.

**Figure S3: Type I Error real-world data simulation results for quasi-Poisson models stratified by percentage of zero counts: A) high, B) medium, C) low**

A)

B)

C)

Footnote: High, medium, and low include 20,000, 40,000, and 30,000 simulation counts, respectively.

**Figure S4: Type I Error real data based simulation results for the count component of ZINB & hurdle negative binomial models stratified by percentage of zero counts: A) high, B) medium, C) low**

A)

B)

C)

ZINB and hurdle models were run for all subsets except CD3^-^ immune cells, due to very low zero counts in that subset (80,000 tests total). High, medium, and low include 20,000, 40,000, and 20,000 simulation counts, respectively. <1% of P-values did not converge for the count component of the hurdle negative binomial models, and all the non-converging models were for the double negative naïve subset. The binary component of the hurdle negative binomial model had no convergence issues. The binary component of the ZINB model, which did not include the exposure of interest and only the area term as a predictor, did not converge 45% of the time, while the count component which did model a simulation of the exposure of interest did not converge <1% of the time.

**Figure S5: Power simulation across different conditions & statistical models under a quadratic NB distribution**

**Figure S6A: Distribution of simulated counts for a high percentage zeros subset under a linear NB distribution**

**Figure S6B: Distribution of simulated counts for a medium percentage zeros subset under a linear NB distribution**

**Figure S6C: Distribution of simulated counts for a low percentage zeros subset under a linear NB distribution**
